# Genetic Algorithms for Feature Selection in the Classification of COVID-19 Patients

**DOI:** 10.3390/bioengineering11090952

**Published:** 2024-09-23

**Authors:** Cosimo Aliani, Eva Rossi, Mateusz Soliński, Piergiorgio Francia, Antonio Lanatà, Teodor Buchner, Leonardo Bocchi

**Affiliations:** 1 Department of Information Engineering, University of Florence, 50139 Florence, Italy; evaros138@gmail.com (E.R.); piergiorgio.francia@unifi.it (P.F.); antonio.lanata@unifi.it (A.L.); leonardo.bocchi@unifi.it (L.B.); 2 School of Biomedical Engineering Imaging Sciences, Faculty of Life Sciences Medicine, King’s College London, London WC2R 2LS, UK; mat.solinski@gmail.com; 3 Engineering Department, Faculty of Natural, Mathematical & Engineering Sciences, King’s College London, London WC2R 2LS, UK; 4 Faculty of Physics, Warsaw University of Technology, Koszykowa 75, 00-662 Warsaw, Poland; teodor.buchner@pw.edu.pl

**Keywords:** heart rate variability, photoplethysmography, COVID-19, genetic algorithms

## Abstract

Background: Severe Acute Respiratory Syndrome CoronaVirus-2 (SARS-CoV-2) infection can cause feared consequences, such as affecting microcirculatory activity. The combined use of HRV analysis, genetic algorithms, and machine learning classifiers can be helpful in better understanding the characteristics of microcirculation that are mainly affected by COVID-19 infection. Methods: This study aimed to verify the presence of microcirculation alterations in patients with COVID-19 infection, performing Heart Rate Variability (HRV) parameters analysis extracted from PhotoPlethysmoGraphy (PPG) signals. The dataset included 97 subjects divided into two groups: healthy (50 subjects) and patients affected by mild-severity COVID-19 (47 subjects). A total of 26 parameters were extracted by the HRV analysis and were investigated using genetic algorithms with three different subject selection methods and five different machine learning classifiers. Results: Three parameters: meanRR, alpha1, and sd2/sd1 were considered significant, combining the results obtained by the genetic algorithm. Finally, machine learning classifications were performed by training classifiers with only those three features. The best result was achieved by the binary Decision Tree classifier, achieving accuracy of 82%, specificity (or precision) of 86%, and sensitivity of 79%. Conclusions: The study’s results highlight the ability to use HRV parameters extraction from PPG signals, combined with genetic algorithms and machine learning classifiers, to determine which features are most helpful in discriminating between healthy and mild-severity COVID-19-affected subjects.

## 1. Introduction

COVID-19 is an infectious respiratory disease caused by SARS-CoV-2, a coronavirus discovered in 2019 [1]. Since then, the virus has spread rapidly around the world, causing a huge global health emergency. According to World Health Organization (WHO) data, more than 770 million cases and more than 6.9 million deaths have been recorded as of September 2023. Although the WHO ended the global public health emergency for COVID-19 on 5 May 2023, this disease is still fearful today [2,3].

In this sense, it is crucial to learn as much as possible, in order to define a useful tool to evaluate, manage, and treat patients, so as to avoid future pandemics with terrible socioeconomic and health experiences. Among the most feared consequences of SARS-CoV-2 infection are the effects on the circulatory and microcirculatory systems. In particular, several studies have reported systemic microcirculatory changes with endothelial dysfunction [4,5] and peripheral nervous system abnormalities [6,7] in COVID-19 patients. The role of endothelial dysfunction is essential, considering that it has been associated with poor prognosis in the acute phase [8] and with persistent symptoms, such as chest pain and fatigue, during the long COVID-19 period (4 weeks or more after onset infection) [9]. Therefore, an analysis of microcirculation may be necessary in both clinical settings and the long-term monitoring of patients’ conditions.

For the timely treatment of patients suffering from diseases such as COVID-19, timely recognition through easy-to-perform and low-cost tests may be particularly important. This may be even more important for controlling costs, which have been unsustainable in some national health systems, and facilitating activity in clinical and laboratory settings. An answer to the need to obtain rapid methods for recognizing patients with COVID-19 can also be obtained from HRV analysis. In this regard, a review of HRV extraction methods from different devices has highlighted that although ElectroCardioGraphy (ECG) devices have served as the gold standard, several alternative devices are more practical for extracting HRV time series [10], mainly based on single-lead ECG and PhotoPlethysmoGraphy (PPG). The PPG technique is a non-invasive, low-cost, and user-friendly method that allows more precise assessment and monitoring of the microcirculation, even remotely, contributing to detecting the disease and its severity [11]. In comparing HRV-related parameters extracted from ECG and PPG, the literature showed that PPG data were accurate enough to detect cardiac rhythm alterations [12].

The literature presents several applications demonstrating the ability of machine learning classifiers to detect COVID-19 infection through PPG signals. In particular, there are both studies in which classifiers were trained using only morphological parameters extracted from PPG signals [13] and in which they were trained using HRV-related parameters [14]. The first one used three different machine learning classifiers—Support Vector Machine (SVM), Bayesian Classifier (BYM), and K-Nearest Neighbor (KNN)—while the second one used Random Forest (RF) and SVM classifiers. Additionally, the ability of HRV-related parameters to be used for COVID-19 detection was also demonstrated when these parameters were extracted from ECG signals [15]: in this study, night-time RR time series were extracted from ECG signals. Some significant differences were found between the parameters considered. Such differences could be caused by changes in the parasympathetic autonomic nervous system activity and by coupling the respiratory rhythm with the heart rate due to increased pulmonary arterial vascular resistance.

Since an integrated approach is necessary to fight the COVID-19 pandemic and others to come [16], the possibility of applying different data analysis solutions, such as genetic algorithms, represents a significant opportunity to study the effects of COVID-19 disease further. The use of genetic algorithms, presented by John Holland [17], allows the simulation of evolution and natural selection processes in genetics, to evaluate different combinations of parameters (genes) that, recombined together with mutations, lead to new solutions (chromosomes) improving fitness function during different iterations. The first step in all genetic algorithms is creating an initial population. This consists of a set of subjects where each individual is represented as a combination of parameters. Each individual is assigned a fitness value that represents its chance of being selected for reproduction, thus passing on its genetic characteristics to its offspring. Subsequently, the selection phase aims to choose the subjects that will become the parents and, thus, transfer their parameters to subsequent generations. This will lead to a child subject represented by a certain number of features given by mixing the parents’ features.

Using genetic algorithms as a feature selection method is well-known in the literature and has been used in many research fields. Navazi et al. [18] showed the effectiveness of using genetic algorithms to select useful features to diagnose type II diabetes mellitus in its early stages, using a public dataset with 17 features. Golap et al. [19] showed how using genetic algorithms can effectively select the best features among 46 extracted from PPG signals, to estimate blood hemoglobin and glucose levels in a non-invasive way. In a study conducted by Miao et al. [20], a wearable sensor collected ECG and PPG signals, and a total of 21 features were extracted, to assess arterial stiffness; finally, a feature selection method based on a genetic algorithm was used to select the important indicators. Interesting studies have also been conducted to evaluate the effectiveness of genetic algorithms for feature selection extracted from medical images. For example, both Albadr et al. [21] and Manav et al. [22] showed the effectiveness of genetic algorithms within a classification process of chest X-ray images for detecting COVID-19.

Given the ability of genetic algorithms to highlight which features within a population have greater relevance, their use could extend beyond purely biomedical fields to more general applications, such as text analysis, sentiment analysis, and document classification using machine learning algorithms [23,24,25,26]. Additionally, the acquisition technique itself could vary: for example, it might be used to enhance segmentation algorithms for ultrasound images [26], or to identify the most relevant wavelet transform coefficients in Laser Doppler Flowmetry (LDF) signals [27].

This study aimed to verify the presence of microcirculation alterations in patients with COVID-19 by performing HRV analysis. In particular, peak-to-peak intervals were extracted from PPG signals. Subsequently, HRV features extraction from these intervals was performed, and an analysis with genetic algorithm and machine learning classifiers was conducted. Genetic algorithms allowed highlighting which extracted features were greatly relevant, to discriminate between healthy and COVID-19 subjects.

## 2. Methods

This study analyzed PPG signals acquired from 97 subjects. In particular, they were selected and divided as follows:

- Group 0 (control group): 50 healthy subjects (age = 45 ± 23 years and male/female ratio = 0.47);
- Group 1 (study group): 47 COVID-19-affected subjects (age = 70 ± 15 years and male/female ratio = 1.61) with P/F>200, RR<20 a/min. and ROX>2.85 at 2 h, ROX>3.47 at 6 h and ROX>3.85 at 12 h. In addition, the subjects required receiving low-flow oxygen therapy (nasal cannula or face masks) or High-Flow Nasal Cannula (HFNC) only without positive pressure ventilatory support. PPG signals were acquired during the infection period.

The Horowitz index P/F or PaO_2_/FiO_2_ was the ratio of arterial oxygen partial pressure (PaO_2_ in mmHg) to fractional inspired oxygen (FiO_2_ expressed as a fraction, not a percentage) [28], RR was the respiratory rate, and the ROX index was expressed as [SpO_2_/FiO_2_]/respiratory rate, where SpO_2_ was the peripheral oxygen saturation [29]. The study group comprised hospitalized patients at San Giuseppe Hospital (Empoli, Italy), while healthy subjects were healthcare operators working in the same hospital. Before every acquisition, each subject signed an informed consent following the Declaration of Helsinki [30]. The local ethical committee, Comitato Etico Regione Toscana—Area Vasta Centro (CEAVC), approved the study protocol (protocol number: 19059).

A PPG signal was acquired from every subject through the commercial monitoring system available in the Intensive Care Unit (ICU) of the hospital. In particular, it was composed of three components: a touchscreen monitor (Mindray ePM 10—Mindray, China), its default finger pulse oximeter, and a data logger to save data (Raspberry Pi 3). In particular, the oximeter was connected to the monitor SpO2 input. These devices were used daily in the hospital, and their proper calibration and validation were certified by the hospital’s clinical engineering department, following standard maintenance procedures. Each enrolled subject underwent an acquisition protocol composed of a 10-min acclimatization phase to avoid data bias and a 5-min acquisition phase. The oximeter was positioned on the right-hand forefinger.

The following sections will describe in detail the processes applied to the PPG signals.

### 2.1. HRV Parameters Extraction

To extract HRV-related parameters from the PPG signals, Python (version: 3.9.13) libraries were used. In particular, these libraries allow the extraction of 26 parameters grouped into three sets: time, frequency, and non-linear features.

The time domain parameters are the following:

- MEAN RR (ms): mean value of RR time intervals;
- STD RR (ms): standard deviation of RR time intervals;
- RMSSD (ms): root mean square between successive RR time intervals differences;
- NN50 (–): the number of times that successive RR time intervals exceed more than 50 ms;
- pNN50 (%): NN50 divided by the total number of RR time intervals;
- NN20 (–): the number of times that successive RR time intervals exceed more than 20 ms;
- pNN20 (%): NN20 divided by the total number of RR time intervals;
- HRV TRIANGULAR INDEX (–): the integral of the RR interval histogram divided by the height of the histogram;

The frequency parameters are divided into different sub-domains based on frequency bandwidth. VLF (Very Low Frequency) includes frequencies in the bandwidth [0.003÷0.04] Hz. It reflects an intrinsic rhythm produced by the heart, which is modulated primarily by the Sympathetic Nervous System (SNS) and from changes in thermoregulation. LF (Low Frequency) includes frequencies in the bandwidth [0.04÷0.15] Hz and reflects a mixture of SNS and Parasympathetic Nervous System (PNS) activity and the baroreceptors’ regulation activity. HF (High Frequency), including frequencies in the bandwidth [0.15÷0.4] Hz, reflects fast changes in beat-to-beat variability due to PNS activity and is also called the “respiratory band” because it corresponds to HRV changes related to the respiratory cycle. It can be increased by slow/deep breathing and decreased by anticholinergic drugs or vagal blockade [31]:

- VLF Power (ms^2^): the absolute power spectrum density of the VLF band;
- LF Power (ms_2_): the absolute power spectrum density of the LF band;
- LF Power (n.u.): the absolute power spectrum of LF band in normalized units. The normalization is defined as LF/(HF + LF);
- HF Power (ms^2^): the absolute power spectrum density of the HF band;
- HF Power (n.u.): the absolute power spectrum of HF band in normalized units. The normalization is defined as HF/(HF + LF);
- LF/HF Power: the ratio between Low-Frequency Power and High-Frequency Power is sometimes used as a quantitative mirror of the SNS/PNS balance.
- Total Power: total power density spectrum.

The non-linear parameters are listed as follows:

- sd1 (ms^2^): the standard deviation of projection of the Poincarè plot on the line perpendicular to the line of identity;
- sd2 (ms^2^): the standard deviation of projection of the Poincarè plot on the line of identity;
- sd2/sd1: ration between sd2 and sd1;
- Sample entropy: provides an estimate of the complexity of a numerical series [32].
- Shannon entropy: is a statistical quantifier extensively used to characterize complex processes. It can detect non-linearity aspects in model series, contributing to a more reliable explanation of the nonlinear dynamics of different analysis points [33].
- pV0, pV1, pLV2, pUV2: Porta’s symbolic parameter related to, respectively, patterns with no variations, patterns with one variation, patterns with two like variations, and patterns with two unlike variations [34];
- alpha1, alpha2: respectively, scaling exponent characterizing short-term correlations (range of n: [4–16]) and scaling exponent characterizing long-term correlations (range of n: [16–64]) [35].

These parameters were then analyzed, using a genetic algorithm to identify which were most informative for discriminating between the two classes of subjects.

### 2.2. Statistical Analysis

Before applying the genetic algorithm to the features, a statistical analysis was conducted to determine which features were statistically different between the Study and Control Groups. Firstly, the Lilliefors normality test was conducted for each parameter [36]. Then, if the parameter in both the Control and Study Groups was determined to be from a normally distributed population, the t-test was employed; otherwise, the Mann–Whitney U test was adopted. A significance level of α=0.05 was applied in both scenarios.

### 2.3. Genetic Algorithm

This section describes the methods for applying the genetic algorithms to the acquired PPG signals. All the parameters analysis and algorithms were implemented in Matlab (version: 9.13.0 (R2022b)). Typically, a genetic algorithm has five phases:

- Initialization: the optimization process begins with an initial population. In particular, every subject within the population has some features.
- Fitness assessment: among the available subjects, it is crucial to select the best ones to reproduce offspring, so each subject is associated with a fitness score, meaning the probability of being chosen during the selection phase.
- Subjects selection: at each iteration, two subjects are selected for the reproduction phase. The idea is to combine the parents’ features to create a new offspring subject.
- Reproduction: generation of offspring subjects usually occurs in two ways, i.e., crossover (mixing the parents’ features) and mutation (applying random features changing).
- Convergence: when there is no improvement in the quality of the solution or after completing a previously established number of iterations, the algorithm is stopped.

**Figure 1:**
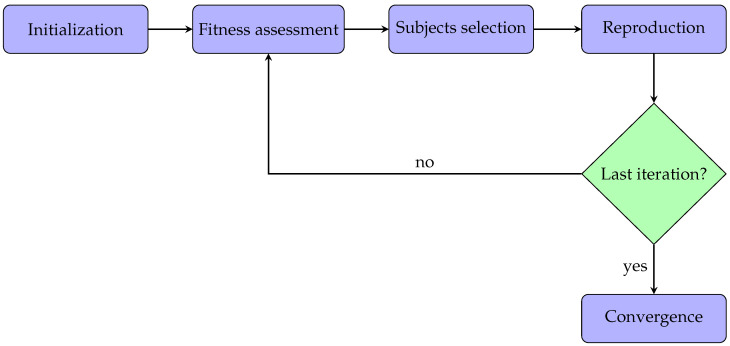
Flowchart diagram related to the application of the genetic algorithm.

In this work, the implemented genetic algorithm was also based on the same five steps described above. Additionally, these phases are represented in the flowchart diagram in Figure 1 for better clarification. Each step will be described and analyzed in detail in the following subsections.

#### 2.3.1. Initialization

Since the aim was to use the genetic algorithm to conduct features analysis, the initial population with which the algorithm was initialized consisted of artificially created subjects More specifically, while each of the 97 subjects in the dataset was associated with 26 HRV-related features, each subject composing the initial population would possess only some of them. In particular, how many and which ones were determined at random.

To assess the impact of the initial population size in detecting the most significant features, four initial populations of different sizes were tested: 6, 16, 26, and 36 subjects. Additionally, to avoid introducing a bias related to the number of subjects in the population (the greater the number of subjects, the greater the probability of having subjects with significant features), populations with more subjects were optimized for a cycles number inversely proportional to the number of subjects:

(1) $$n_{c}=1000\frac{6}{N_{p}}$$

where $N_{p}$ represented the number of the initial population (6, 16, 26, and 36) and $n_{c}$ was the number of optimization cycles for that specific population. Thus, the number of optimization cycles $n_{c}$ with an initial population size $N_{p}$ of 6, 16, 26, and 36 were, respectively: 1000, 375, 231, and 167.

Additionally, as each initial population consisted of subjects with randomly chosen features, to avoid as much as possible a bias introduced by their randomness, each application of the genetic algorithm with the four different initial populations was repeated 5 times. The performance of the genetic algorithms on each population was obtained by averaging the results over the five iterations.

#### 2.3.2. Fitness Assessment

Each subject belonging to the initial population was characterized by some features among those initially extracted from the PPG signals. The following procedure was used to assess the fitness score for each subject:

- Features transfer: if the subject being evaluated possessed only certain specific features, the initial dataset consisting of 97 subjects was processed in such a way that each subject within it contained only these features;
- Accuracy evaluation: the accuracy of different machine learning classifiers in discriminating between the two classes (Group 0 and 1), once trained with those features only, was assessed.

Specifically, the training and testing policy adopted during the accuracy evaluation phase was Leave One Subject Out (LOSO), which consists of removing a subject from the dataset (composed of *N* subjects), training the classifier on the remaining *N-1* subjects, and testing on the removed subject. Afterwards, the removed subject is reinserted into the dataset, and the steps are repeated iteratively until all the *N* subjects have been removed once. The global performance of the classifier for that particular subject is evaluated by averaging the performance of every single iteration. This value is then assigned as the fitness value of the tested subject.

In this study, five different supervised classifiers were tested: K-Nearest Neighbor (KNN), binary Decision Tree (DT), discriminant analysis classification (DISCR), Naive Bayes (NB), and logistic regression model (LOGIT).

#### 2.3.3. Selection Phase

Three different criteria for selecting and improving individuals’ fitness were used:

- Two-random-subjects selection method: within the population, two subjects, $S_{1}$ and $S_{2}$, are chosen randomly. They generate two new subjects, $S_{1-2}$ and $S_{2-1}$, that take the place of $S_{1}$ and $S_{2}$ within the population, so that the total number of individuals remains equal to *N*; then, the cycle starts again. This method is the least robust because the individuals are randomly chosen and not fitness-based chosen.
- Five-subjects tournament selection method: within the population, five subjects are selected randomly and sorted in ascending order, concerning the fitness value. Then, the first two, in terms of fitness, are selected, and two new subjects are created. This procedure is repeated until a new population consisting exclusively of new subjects of the same size as the starting population has been created; then, the cycle starts again.
- Roulette wheel selection method: the fitness value of each subject belonging to the population is evaluated. Then, two subjects are selected, based on the subjects’ fitness value: subjects with a greater fitness value will have a greater chance of being chosen than subjects with a smaller one. Finally, two new subjects, $S_{1-2}$ and $S_{2-1}$, are created. This procedure is repeated until a new population, consisting exclusively of new subjects of the same size as the starting population, has been created; then, the cycle starts again.

#### 2.3.4. Reproduction Phase

The reproduction phase is a fundamental step of genetic algorithms. In this study, once two subjects had been chosen, using one of the previous methods, the generation phase consisted of creating two new subjects. This phase was composed of two sub-phases, known as crossover and mutation:

- Crossover: once subjects $S_{1}$ and $S_{2}$ were chosen within the population, their features were mixed. In particular, random features from subject $S_{1}$ were assigned to subject $S_{2}$, leading to the creation of two new subjects, $S_{1-2-A}$ and $S_{1-2-B}$. This process was then inverted, by assigning random features from subject $S_{2}$ to subject $S_{1}$, and so creating subjects $S_{2-1-A}$ and $S_{2-1-B}$. Finally, only the two subjects with the greater fitness value were maintained.
- Mutation: the mutation phase was applied, using a mutation coefficient of 0.5, i.e., each of the two maintained subjects had a 50% chance of undergoing random features mutations. This meant that there was a possibility of some features in that subject being replaced by others or being added or removed.

### 2.4. Data Extraction

The following parameters were calculated at each optimization cycle:

- Mean fitness: represented how much, on average, that population comprised individuals with significant features.
- Std fitness: represented fitness-level variability within the population.
- Best fitness: represented the highest fitness value within that population.
- Features of the best five subjects: represented the features present in the five subjects with the highest fitness.

## 3. Results

Table 1 shows the results of the statistical analysis.

Table 2, Table 3 and Table 4 report the mean, standard deviation, and maximum fitness values for each subject selection method, machine learning classifiers, and initial population size.

**Figure 2:**
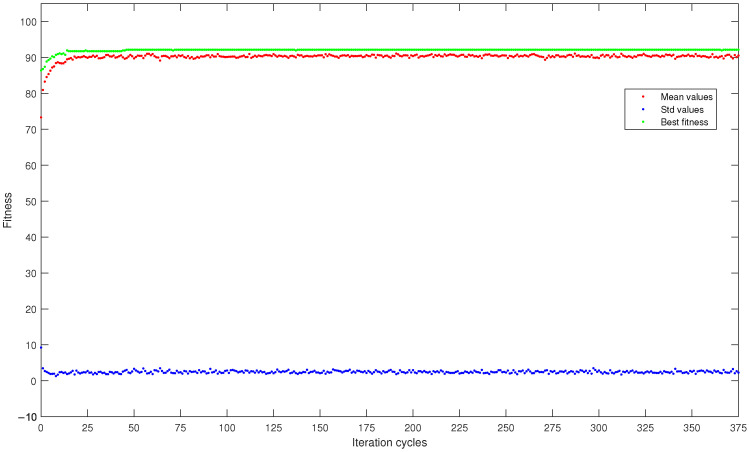
Curves, averaged over the five runs, of mean fitness (red dots), maximum fitness (green dots), and standard deviation (blue dots) within the population consisting of 16 subjects and analyzed by the DISCR classifier with the roulette wheel selection method.

To better analyze the progress of the fitness refinement, Figure 2 reports a graph showing mean fitness values, maximum fitness, and standard deviation of fitness within the population as the optimization cycles changed, all averaged over the five runs. In particular, it is related to the best result in terms of fitness value obtained in this study: the DISCR classifier, initial population size of 16 subjects, with the roulette wheel selection method.

**Figure 3:**
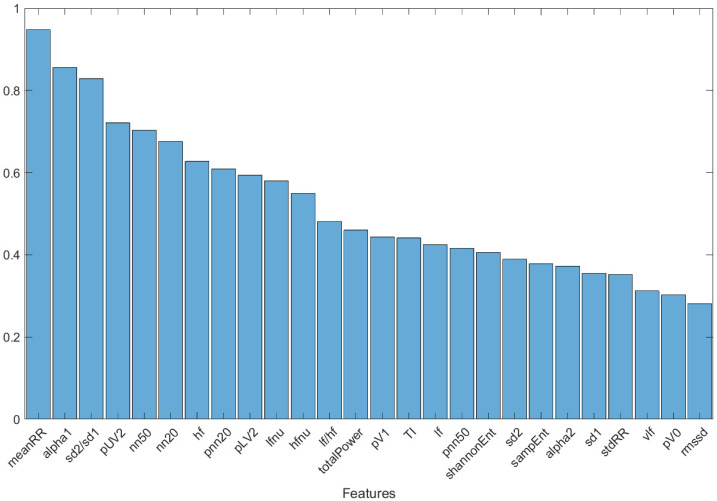
Histogram of the features of the five subjects with the highest fitness value over the last 50 optimization cycles, including all selection methods and classifiers used.

To assess which features had the greatest ability to discriminate between the two classes of subjects (Group 0 and Group 1), a graph was created (Figure 3) showing the features of the five subjects with the greatest fitness value over the last 50 optimization cycles, including all the selection methods, classifiers, and different starting population sizes. A combining of the results independently from the selection methods, classifiers, and starting population sizes was undertaken, to highlight those features considered fundamental overall. The decision to report features restricted to the last 50 optimization cycles was motivated by the stability of the result (see Figure 2): this avoided the whole first transitory part, where the population fitness value stabilized and optimized. The graph reported in Figure 3 is normalized, concerning the maximum value.

Finally, to validate the results of using genetic algorithms as feature selection methods, classification was carried out by training the same five classifiers with the three most relevant parameters shown in Figure 3: meanRR, alpha1, and sd2/sd1. The results obtained when the machine learning classifiers were trained with either all HRV parameters or only the three most significant ones are shown in Table 5.

As reported in Table 5, the best result was obtained with the DT classifier trained with only the three most relevant features, achieving accuracy of 82%, specificity (or precision) of 86%, and sensitivity of 79%.

**Figure 4:**
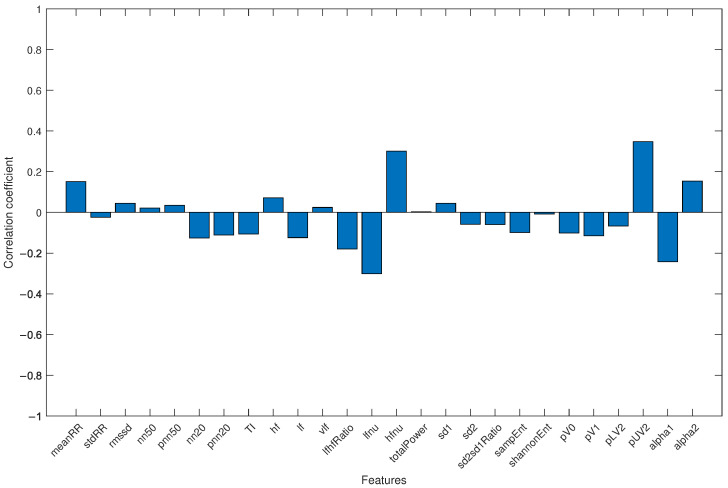
Correlation coefficient for each HRV feature and age when the study group and control group were jointly considered.

Finally, to avoid the results being influenced by the different average ages of the two groups (with healthy subjects being younger than COVID-19 patients), all subjects were merged into a single group, and the correlation coefficient between each HRV parameter and age was analyzed. The result is shown in Figure 4.

## 4. Discussions

The statistical differences observed in the parameters reported in Table 1 suggest that COVID-19 significantly affects microcirculation, leading to alterations in HRV parameters. In fact, among the 26 HRV-related parameters, 17 were statistically different between the two groups: 10 with p<0.05 and 7 with p<0.01. This could suggest that COVID-19 significantly affects microcirculation, leading to alterations in HRV-related parameters.

The results reported in Table 2, Table 3 and Table 4 highlight the ability of the three genetic algorithm selection methods to determine which features could be more useful in discriminating between control and study groups.

Focusing on the initial (start) and final (end) fitness values, the different classifiers and methods were all capable of increasing the mean fitness value and decreasing the variability during the optimization process. This underscores the ability of the proposed genetic algorithm to self-determine, during the optimization rounds, which features are most useful in discriminating between healthy (Group 0) and mild-severity COVID-19 subjects (Group 1).

It is also noticeable that for the same subject selection method and classifier, the achieved maximum fitness value was almost invariant from the number of subjects comprising the initial population. This may suggest that the discriminatory abilities of the tested classifiers did not depend on the initial number of subjects. In other words, each classifier had a better or worse ability to discriminate between subjects in Group 0 and Group 1, and this ability did not depend on how many such subjects composed the initial population. This may suggest that in future research studies adopting genetic algorithms, the initial population with which the algorithm is initialized could be kept low, as not being capable of significantly affecting the final result.

Additionally, the fitness end value remained approximately the same among all the three subjects’ selection methods with the same classifier but changed with different classifiers. This may suggest that it is not important how subjects are chosen within a population but, instead, which classifier is used to assess their fitness value.

It can also be seen that the DISCR classifier was the one that, for each subject’s selection method, achieved the best results, in terms of maximum fitness value within the population: 91.8 for both the two-random-subjects and five-subjects tournament selection methods and 92.2 with the roulette wheel selection method. In particular, the absolute best performance, with a final maximum fitness value of 92.2, was achieved using the roulette wheel method with an initial population size of 16 subjects. This may suggest that the assumptions and analysis methods adopted by the DISCR classifier are more suitable, compared to the other tested classifiers, for the analysis of HRV data in the COVID-19 context.

Regarding the results obtained by the LOGIT classifier, it can be seen from Table 2, Table 3 and Table 4 that the fitness values achieved were consistently in line with, although often lower than, those of the DISCR classifier. This suggests that even a model based on logistic regression (a linear combination of multiple independent variables) could be a suitable model for the analysis of HRV data in the context of COVID-19.

Focusing on Table 2, it can be seen that the two-random-subjects selection method was the one that achieved slightly lower fitness values. Despite this, an improvement was still noticeable, as during new subjects’ creation only those with higher fitness values were maintained. This suggests that a random subject selection method could be a valid choice as an initial approach, due to its simplicity and speed, but that, to achieve better results, fitness-based subject selection methods seem more suitable and, therefore, more robust.

Moving to Figure 2, the mean and maximum fitness values within the population followed an increasing trend as the iterations increased, starting from values of 73.3 and 86.4, respectively, and reaching higher values of 90.5 and 92.2. In contrast, the fitness standard deviation showed an opposite trend, decreasing as the iterations increased. In particular, it decreased from an initial value of 9.2 to a final value of 2.3. These increasing and decreasing trends were also obtained for all the other subject selection methods, independently from which machine learning classifiers and initial population size were used. The fact that a constant trend was achieved almost immediately indicates that future research studies adopting genetic algorithms could reduce the maximum number of iterations, making the analysis process faster.

Finally, the histogram in Figure 3 shows the features of the five subjects with the greatest fitness value over the last 50 optimization cycles, including all the subject selection methods, machine learning classifiers, and initial population sizes. Three predominant features emerged: *meanRR*, *alpha1*, and *sd2/sd1*. These features were present, respectively, in 94.2%, 80.2%, and 78% of the subjects. The importance of these three parameters was confirmed by the classification results performed with the DT classifier trained and tested with them only. The accuracy of 82% confirmed the strong information content of these three parameters in the discrimination between healthy and mild-severity COVID-19-affected subjects, using HRV-related parameters.

The differences in these parameters between the groups shown in Table 1, as well as for other features not highlighted by the proposed genetic algorithm (parameters in the frequency domain, pUV2), could be an effect of changes in the Parasympathetic Nervous System. These results are coherent with the previous findings reported in comparison studies between healthy individuals and COVID-19 patients, either during infection [37,38] or recovery period [39]. Additionally, a during-infection study suggested that the changes could be related to increased peripheral resistance in pulmonary circulation due to SARS-CoV-2 [15]. An increase in parasympathetic activity could have been responsible for the higher meanRR parameter value in the study group. This is also supported by the RMSSD parameter: the higher RMSSD values in the study group suggest that an increase in vagal tone can be observed in COVID-19 patients, despite their older age. In the study by Maartje et al. [40], the authors suggested that the vagus nerve appears to inform the brain about peripheral inflammation. As a result, cortisol production is activated via the hypothalamic–pituitary–adrenal axis. The authors also highlighted that, due to descending efferent vagal-to-sympathetic neural conversion, a subclass of T-cells secretes acetylcholine, limiting the production of inflammatory cytokines by splenic macrophages. When vagal activity is low, the inflammatory response can escalate into a “cytokine storm”.

The second highlighted parameter, alpha1, was previously reported as a relevant biomarker of the inflammatory process and was connected with depression [41]. A decrease in that parameter was correlated with white blood cell count and fibrinogen level. Finally, regarding the sd2/sd1 parameter, some similarities between the presented results and the outcomes of a risk prediction model for septic patients with Systemic Inflammatory Response Syndrome (SIRS) [42] were found. In particular, the HRV-related parameters analysis in a patients group with 30-day In-Hospital Mortality (30-day IHM) had a higher meanRR and a lower sd2/sd1 ratio compared to the no 30-day IHM group. The top 10 predictive features in the gradient boosting model included, among other things, HRV-related parameters selected by our algorithm (or their relatives): alpha1 and meanRR.

Regarding the fact that the study group was significantly older than the control group, differences in HRV-related parameters for different age groups are well-documented in the literature [43,44]. It has also been observed that either the mean RR interval or HRV decrease with age, due to changes in short-term HRV-related parameters in all three domains (time, frequency, and non-linear). In particular, a reduction of SDNN, RMSSD, pNN50, LF, HF, totalPower, and increased Detrended Fluctuation Analysis (DFA) scaling factors were highlighted [45]. The results obtained in this work show an inverse relationship between the mean values of HRV-related parameters and age (see Table 1), and this observation also applies to the parameters highlighted by the genetic algorithm. This may indicate that the differences between the groups are not due to the difference in mean age. A further confirmation is provided by the evaluation of the correlation coefficient between each extracted HRV feature and age (see Figure 4): the correlation coefficients were always lower than 0.35, emphasizing the fact that the results were not influenced by the age difference between groups.

Despite the results achieved, there was a limitation. The dataset studied, consisting of 97 subjects with demographic discrepancies, could potentially affect the generalizability of the results. Further studies conducted on a larger sample of subjects with more consistent demographic characteristics could better validate the findings.

## 5. Conclusions

- This study demonstrates the efficacy of the described genetic algorithm in identifying key HRV-related features, extracted from PPG signals, for discriminating between control and study groups using machine learning classifiers.
- The consistent fitness end value across different subject selection methods and machine learning classifiers implies that the choice of classifier significantly influences fitness assessment, rather than how subjects are chosen within the population.
- Three features, meanRR, alpha1, and sd2/sd1, were revealed by the genetic algorithm as pivotal in distinguishing between healthy individuals and COVID-19 patients with mild disease severity.
- A binary Decision Tree classifier, trained and tested with these three parameters only, achieved 82% accuracy, demonstrating strong discriminatory power.
- The differences in HRV parameters, particularly those related to the Parasympathetic Nervous System, between the control and study groups aligned with the existing literature on COVID-19 patients’ physiological changes during infection and recovery. Furthermore, similarities with risk prediction models for other medical conditions, such as sepsis, underscore the clinical relevance of the findings.
- Since the PPG signal is commonly acquired and used in clinical practice, the described methodology could represent the first step towards developing more targeted, patient-specific approaches for diagnosis and monitoring.

## Figures and Tables

**Table 1:** Mean and standard deviation of the parameters in the control group (Group 0) and study group (Group 1) grouped for each set: time, frequency, and non-linear. The symbol “*” indicates statistically significant differences with p<0.05, while the symbol “**” indicates statistically significant differences with p<0.001.

Parameter	Control	Study	*p*-Value
meanRR	796.3±114	864.1±147.5	*
stdRR	69.3±26.2	75.4±84.1	0.058
rmssd	59.8±31.5	75.5±100.4	0.31
nn50	34.1±24.6	35.5±48.8	*
pnn50	15.6±12.1	18.7±21.9	0.46
nn20	113.8±42.3	82.3±55.8	**
pnn20	48.8±13.4	44.6±21.8	0.253
TI	7.1±2.3	5.87±3.1	*
hf	772.4±776.5	1713±4712	*
lf	1937.2±1801	1650±3581	**
vlf	844.9±697.3	2306±8201	0.17
lf/hf	3.4±2.4	2.5±3.6	**
lfnu	70.7±14.3	56.7±20.1	**
hfnu	29.3±14.3	43.3±20.1	**
totalPower	3554±2744	5669±13,874	*
sd1	42.4±22.3	53.6±71.2	0.31
sd2	87.6±31.8	90±97.4	*
sd2/sd1	2.3±0.6	2±1.1	*
sampEnt	1.6±0.4	1.4±0.5	*
shannonEnt	7.8±0.7	7.4±0.6	*
pV0	40.9±21.6	35.5±22	0.09
pV1	38.6±12.1	34.5±10.7	*
pLV2	6.3±5.4	5.8±5.2	0.71
pUV2	14.2±8.3	24.3±11.6	**
alpha1	1.16±0.16	0.91±0.4	**
alpha2	0.84±0.23	0.95±0.41	0.1

**Table 2:** Mean fitness and standard deviation values for each tested classifier, using the two-random-subjects selection method. The table shows the fitness values related to the initial population (start) and to the last optimization cycle (end). The maximum reached values are shown in bold type. All the reported values are the averaging over the five iterations.

Population	Fitness	KNN		DT		DISCR		NB		LOGIT	
6	Start	65 ± 8	**75.9**	74.6 ± 8.7	**83.7**	73.6 ± 6.7	**83.5**	63.7 ± 5.2	**70.9**	76.9 ± 5	**84.1**
	End	79.8 ± 3.9	**81.9**	87.5 ± 2	**88.9**	87.4 ± 1.3	**89.1**	84.7 ± 2.3	**86.4**	87.2 ± 2.1	**89.1**
16	Start	67.7 ± 7.6	**79.6**	75.4 ± 8.1	**86.8**	73 ± 8.4	**84.3**	64.7 ± 5.8	**75.3**	73.3 ± 7.3	**84.3**
	End	81.2 ± 2.4	**82.3**	88.5 ± 1.6	**89.3**	88 ± 2.5	**90.7**	85.3 ± 3	**88.5**	87.6 ± 2.4	**90.1**
26	Start	66.3 ± 7.7	**80**	74.6 ± 8.3	**86.2**	72.9 ± 8.6	**85.8**	64.7 ± 5.8	**76.9**	72 ± 9.3	**84.3**
	End	81.2 ± 2.8	**82.3**	88 ± 2	**88.9**	88.8 ± 2	**91.3**	84.9 ± 3	**88.5**	87.6 ± 2.3	**90.5**
36	Start	67.2 ± 6.9	**80.8**	74.5 ± 8.3	**87.2**	73.1 ± 9.3	**85.6**	65 ± 6	**78.1**	70.9 ± 10.2	**84.5**
	End	81.7 ± 2.5	**82.5**	88 ± 2	**88.7**	89 ± 1.9	**91.8**	85.6 ± 3.3	**89.3**	88.6 ± 2	**91.3**

**Table 3:** Mean fitness and standard deviation values for each tested classifier using the five-subject tournament selection method. The table shows the fitness values related to the initial population (start) and to the last optimization cycle (end). In bold type are shown the maximum reached values. All the reported values are the averaging over the five iterations.

Population	Fitness	KNN		DT		DISCR		NB		LOGIT	
6	Start	69.7 ± 7.2	**79.6**	76.4 ± 8.8	**84.7**	71 ± 9	**80.8**	64.1 ± 4.9	**70.9**	72.7 ± 8.7	**81.2**
	End	82.4 ± 1.5	**83.1**	89.8 ± 1	**90.3**	90.1 ± 2	**91.3**	87.5 ± 2.8	**89.3**	89.6 ± 2.9	**91.5**
16	Start	68.4 ± 7.6	**81**	75.3 ± 7.2	**84.5**	72.9 ± 8.1	**83.7**	64.6 ± 5.6	**75.9**	72.1 ± 9.4	**83.5**
	End	81.6 ± 2.1	**82.5**	90.6 ± 1.2	**91.1**	89.9 ± 1.9	**91.3**	88.2 ± 2.1	**89.5**	89.9 ± 2.6	**91.3**
26	Start	67.4 ± 7	**81**	75.7 ± 7.9	**85.6**	74.3 ± 7.6	**85.8**	64.5 ± 5.2	**75.1**	71.3 ± 7.8	**82.7**
	End	81.3 ± 3.2	**82.3**	87.6 ± 2.6	**88.7**	90.4 ± 2.4	**91.8**	87.5 ± 3.6	**89.5**	89.9 ± 2.7	**91.3**
36	Start	67.2 ± 7.2	**80.6**	73.9 ± 9	**86.6**	73.6 ± 8.7	**85.2**	64.4 ± 5.5	**78.1**	72.2 ± 8.5	**83.3**
	End	81.5 ± 3.3	**82.5**	89.8 ± 2.6	**90.7**	89.7 ± 2.9	**91.5**	87.7 ± 3.5	**89.7**	90.4 ± 2.5	**91.8**

**Table 4:** Mean fitness and standard deviation values for each tested classifier, using the roulette wheel selection method. The table shows fitness values related to the initial population (start) and to the last optimization cycle (end). The maximum reached values are shown in bold type. All the reported values are the averaging over the five iterations.

Population	Fitness	KNN		DT		DISCR		NB		LOGIT	
6	Start	64.9 ± 8.5	**75.1**	75.1 ± 8.8	**84.9**	71.2 ± 7.1	**79.6**	63.4 ± 5.5	**70.1**	71.6 ± 8.8	**81.9**
	End	81.3 ± 1.7	**82.1**	87.9 ± 1.3	**88.7**	89.6 ± 2.9	**91.8**	86.3 ± 2.9	**88.2**	89.2 ± 2	**90.7**
16	Start	66.9 ± 7	**79.4**	75.6 ± 7.3	**86.2**	73.3 ± 9.2	**86.4**	65.1 ± 5.6	**75.9**	71.5 ± 7.9	**82.9**
	End	82.1 ± 1.3	**82.5**	88.8 ± 1.7	**89.5**	90.5 ± 2.3	**92.2**	86.8 ± 3.7	**89.3**	90.1 ± 1.6	**91.1**
26	Start	66.8 ± 7.4	**80.2**	73.6 ± 9.3	**85.6**	74 ± 7.8	**86**	64.3 ± 5.1	**76.3**	71.7 ± 8.6	**84.9**
	End	81.8 ± 2.9	**82.5**	88.9 ± 2	**89.7**	90 ± 2.3	**91.5**	87.1 ± 3	**89.3**	89.8 ± 2.6	**91.5**
36	Start	67.8 ± 7.8	**81.9**	74.2 ± 8.7	**87**	73.3 ± 8.5	**87**	65.1 ± 5.9	**76.7**	71.7 ± 8.8	**83.9**
	End	81.5 ± 3.1	**82.5**	89.1 ± 1.4	**89.5**	89.9 ± 2.6	**91.5**	87.4 ± 3	**89.5**	90.1 ± 2.7	**91.8**

**Table 5:** Results of machine learning classifiers expressed as accuracy, specificity, and sensitivity. Values in parentheses represent the results when the classifiers were trained and tested using only the three most relevant parameters: meanRR, alpha1, and sd2/sd1.

Classifier	n° of Features	Accuracy	Specificity	Sensibility
KNN	All (3)	75 (45)	76 (50)	74 (40)
DT	All (3)	80 (82)	84 (86)	77 (79)
DISCR	All (3)	82 (75)	80 (80)	85 (87)
NB	All (3)	65 (78)	90 (86)	38 (70)
LOGIT	All (3)	78 (79)	80 (81)	76 (77)

## Data Availability

The datasets presented in this article are not readily available, due to privacy limitations.

## Nomenclature

HRV	Heart Rate Variability
ECG	ElectroCardioGraphy
PPG	PhotoPlethysmoGraphy
Laser Doppler Flowmetry	(LDF)
VLF	Very Low Frequency
LF	Low Frequency
HF	High Frequency
SNS	Sympathetic Nervous System
PNS	Parasympathetic Nervous System
LOSO	Leave One Subject Out
DISCR	Discriminant analysis classification
DT	Decision Tree
KNN	K-Nearest Neighbor
NB	Naive Bayes
LOGIT	LOGIsTic regression model
